# Older adults at high risk of HIV infection in China: a systematic review and meta-analysis of observational studies

**DOI:** 10.7717/peerj.9731

**Published:** 2020-10-21

**Authors:** Yuan Yuan Wang, Yuan Yang, Chang Chen, Ling Zhang, Chee H. Ng, Gabor S. Ungvari, Xiaohua Douglas Zhang, Yu-Tao Xiang

**Affiliations:** 1 Faculty of Health and Life Sciences, De Montfort University, Leicester, United Kingdom; 2 Center for Cognition and Brain Sciences, University of Macau, University of Macau, Macao SAR, China; 3 Department of Psychiatry and Psychology, Southern Medical University Nanfang Hospital, Guangdong, China; 4 The National Clinical Research Center for Mental Disorders & Beijing Key Laboratory of Mental Disorders & the Advanced Innovation Center for Human Brain Protection, Beijing Anding Hospital, School of Mental Health, Capital Medical University, Beijing, China; 5 Department of Psychiatry, The Melbourne Clinic and St Vincent’s Hospital, University of Melbourne, Richmond, Victoria, Australia; 6 University of Notre Dame Australia, Perth, Fremantle, Australia; 7 Division of Psychiatry, Medical School, University of Western Australia, Perth, Australia

**Keywords:** HIV/AIDS, Older adults, China, Meta-analysis

## Abstract

There is an increasing prevalence of human immunodeficiency virus (HIV) infection in older adults in China, but the findings across prevalence studies have been mixed. This is the first meta-analysis of the prevalence of HIV infection and its moderating factors in older adults in China. Two investigators systematically and independently searched both international (PubMed, PsycINFO, Web of Sciences and EMBASE) and Chinese (WanFang, CNKI, and CQVIP) databases. HIV infection rates in older adults were analyzed using the random-effects model. Altogether 46 studies were included in the analysis. The pooled prevalence of HIV infection in older adults was 2.1% (95% CI [1.9%–2.3%], *I*^2^ = 99.4%). Subgroup analyses revealed that men who have sex with men (MSM), hospital population samples, publications after 2014, studies conducted in the western region of China, and higher study quality were significantly associated with higher HIV infection rate. This meta-analysis found that the HIV infection prevalence in older adults is significantly higher than the general population in China. Attention should be given to this urgent public health issue, and effective HIV/AIDS preventive, screening and treatment measures are warranted in this population. PROSPERO: CRD42019124286.

## Introduction

Human Immunodeficiency Virus (HIV) infection is a major public health challenge associated with high disease burden globally (Maartens, Celum & Lewin, 2014). In 2017, approximately 36.9 million people lived with HIV infection worldwide (UNAIDS, 2019). In China, the incidence of HIV was 4.1 per 100,000 individuals in 2018, which translates to approximately 56,993 newly infected persons (National Bureau of Statistics of China, 2018). Those who are at high risk of HIV infection include men who have sex with men (MSM) and younger adults (Knudson et al., 2018; Zhang et al., 2017b). In China, the highest HIV infection rate occurs in those aged between 20 and 40 years (Lu et al., 2008), but there has been an increasing prevalence in older adults recently, which is similar to trends in Western countries. For instance, around half of the people living with HIV were above 50 years old in the USA (High et al., 2012; Hosaka et al., 2019). In Europe, the number of adults aged 50 years or above living with HIV has been also growing (Seeley, 2017). A study in Chongqing, China found that the number of newly HIV infected persons aged over 50 years has increased six-fold from 1,091 in 2004 to 6,352 in 2015 (Zhang, Rongrong & Guohui, 2017a).

Epidemiological studies in China has found an increasing trend in the number and proportion of HIV infection among older adults (Xing et al., 2014). In the past decades, insufficient attention has been paid to older adults with HIV infection, with a lack of HIV risk reduction interventions (Cooperman, Arnsten & Klein, 2007; Crystal et al., 2003). Knowledge of safe sex activities and condom use are usually not addressed in older adults (Negin & Cumming, 2010), even though high risk sexual behaviors exist in this population (Crystal et al., 2003). In China, neglected sex education among older adults have been associated with unprotected sexual activities (Xing et al., 2014), which could increase the risk of HIV infection. On the other hand, the change in the age distribution of HIV infection in China could be partly due to the widespread use of free Highly Active Antiretroviral Therapy (HAART) in reducing transmission risk of HIV infection (Bhaskaran et al., 2008) among younger adults. Greater access to HIV clinics, free HAART treatment, and improved general healthcare service system in China have also increased their life expectancy therefore most are living to older adulthood (Greenbaum et al., 2008; Xing et al., 2014). These factors may also contribute to the growing proportion of older adults with HIV infection.

In order to develop appropriate strategies for HIV prevention and control, it is important to accurately determine the prevalence of HIV infection across different age groups. To date, no meta-analysis or systematic review of the prevalence of HIV infection in older Chinese adults has been published, which gave us impetus to conduct a meta-analysis to examine the prevalence and its associated factors in this population. In addition, we examined the association of demographic and clinical characteristics, such as sex and sexual orientation, with the prevalence of HIV infection in older adults using subgroup and meta-regression analyses. The target demographic and clinical characteristics were selected based on previous studies (Knudson et al., 2018; Zhang et al., 2017b).

## Methods

### Search strategy and selection criteria

This meta-analysis was performed according to the Preferred Reporting Items for Systematic Review and Meta-Analyses (PRISMA) (Moher et al., 2009). Both International (PubMed, PsycINFO, Web of Science, and EMBASE) and Chinese (WanFang, CNKI and CQVIP) databases were systematically and independently searched by two investigators (Yuan Yang and Chang Chen) from their inception date up to May 1, 2020. The following search terms were used: (“HIV” OR “AIDS” OR “human immunodeficiency virus” OR “acquired immune deficiency syndrome”) AND (“epidemiology” OR “prevalence” OR “rate” OR “proportion”) AND (“old” OR “older” OR “elderly” OR “aged” OR “aging”) AND (“Chinese” OR “China”). Reference lists of eligible studies and relevant review articles were also hand-searched.

The inclusion criteria were studies that: (1) reported prevalence of HIV infection in older adults or provided information to calculate the prevalence. The diagnosis of HIV infection was based on study-defined criteria; (2) were cross-sectional or cohort studies (only baseline data were included) with meta-analyzable data; (3) were conducted in older adults (i.e., aged 50 years and older) in China. Case series, reviews, and meta-analyses were excluded.

### Outcome measures

The outcome measure of the meta-analysis was the prevalence of HIV infection in older adults. For each study, the prevalence was calculated by the number of HIV-infected older adults divided by the total number of older adults.

### Data extraction

Systematic literature search and data extraction were independently conducted by the same two investigators. The titles and abstracts of potential publications were screened separately by the same two investigators before the full texts were read for eligibility. Any inconsistencies in the process were discussed and resolved by a third reviewer (Yuan-Yuan Wang). The following information was extracted: year of publication, survey period, study site, sampling method, sample size and response rate, mean age, sex, education, occupation, province, rural or urban area, definition of older adults (e.g., above 50 years), and transmission route (e.g., commercial sex).

### Quality assessment

The same two investigators independently evaluated the methodological assessment, using the critical appraisal for epidemiological studies (Loney et al., 1998) that contains 8 items covering three aspects: sampling, measurement and analysis. The total score of this instrument is 8; the total score of 7–8 was considered as ‘high quality’, 4–6 as ‘moderate quality’, and 0–3 as ‘low quality’ (Loney et al., 1998). Any inconsistencies were resolved by a discussion with a third investigator.

### Data analysis

The Comprehensive Meta-Analysis software, Version 2.0 (http://www.meta-analysis.com/) and Open Meta-Analyst (http://www.cebm.brown.edu/openmeta/) were used to synthesize data (DerSimonian & Laird, 1986) in all the meta-analytic outcomes. Due to different sampling methods, study designs and demographic and clinical characteristics between studies, the random-effects model was used (DerSimonian & Laird, 1986). The heterogeneity of outcomes were assessed using *I*^2^, with *I*^2^ > 50% as significant heterogeneity (Higgins et al., 2003). Following the recommendation of the Cochrane handbook (Higgins et al., 2019) and other studies (Li et al., 2020; Xu et al., 2020; Yang et al., 2020), publication bias was assessed using the funnel plots and Begg’s test and Tweedie’s trim-and-fill analysis. All data analyses were 2 tailed, and the significant level was set at 0.05.

Subgroup and meta-regression analyses were performed to examine the moderating factors of HIV infection prevalence. Subgroup analyses were conducted according to the following categorical variables: (1) sexual orientation: MSM vs. not specified; (2) sex: male predominance (male percentage ≥ 60%) vs. no-predominance; (3) study site: hospital vs. community; (4) route of transmission: commercial sex vs. non-specified; (5) education: equal to and below primary school vs. above primary school education; (6) occupation: predominance of farmers (i.e., farmer percentage ≥ 60%) vs. no such predominance; (7) economic region: western vs. middle of vs. eastern region of China; (8) area: rural vs. urban; and (9) cut-off age for older adults: ≥50 years vs. ≥60 years, and (10) publication year: during and before 2014 vs. after 2014 (using median splitting method). Meta-regression analyses were conducted to examine the moderating effects of continuous variables (such as quality assessment score and study period) on the results when the number of included studies was at least 10.

## Results

### Literature search and study characteristics

**Figure 1:**
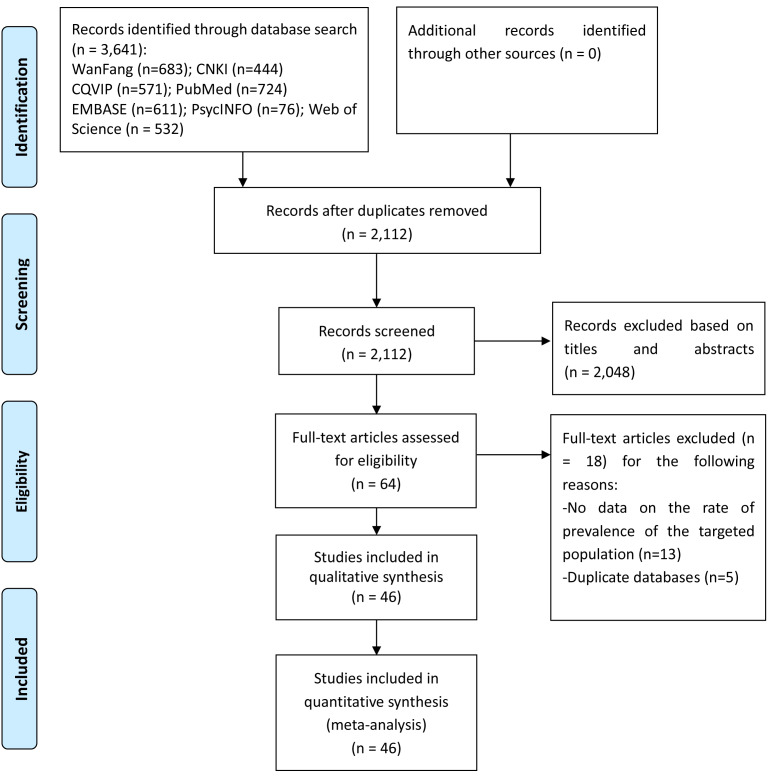
PRISMA flow diagram.

A total of 3,641 publications were identified in the initial literature search. Finally, 46 studies with 363,399 subjects met the entry criteria and were included in the meta-analysis (Fig. 1); four studies were published in English (Chen et al., 2016; Feng et al., 2009; Ning et al., 2018; Xie et al., 2014), and the remaining were published in Chinese language. Table 1 shows the study characteristics. All studies were published between 2004 and 2018. Most studies (89%) used ≥ 50 years as cut-off age for older adults. Of the 46 studies, only one reported treatment information in HIV infected older adults, i.e., 53.75% of whom had HAART treatment (Ning et al., 2018).

Nine studies reported the HIV infection rate in all age groups (Chen et al., 2017; Chen et al., 2016; Chen et al., 2013; Du & Meng, 2016; Feng et al., 2009; Wang et al., 2018a; Zhao, Su & Jiang, 2015; Zhong et al., 2017; Zhu, Miu & Zhang, 2014), therefore only data in older adults were extracted for analyses; while five studies focused on older MSM adults. Thirteen studies reported transmission routes, while the remaining (71.7%) did not.

### Prevalence of HIV infection in older adults and moderating factors

**Figure 2:**
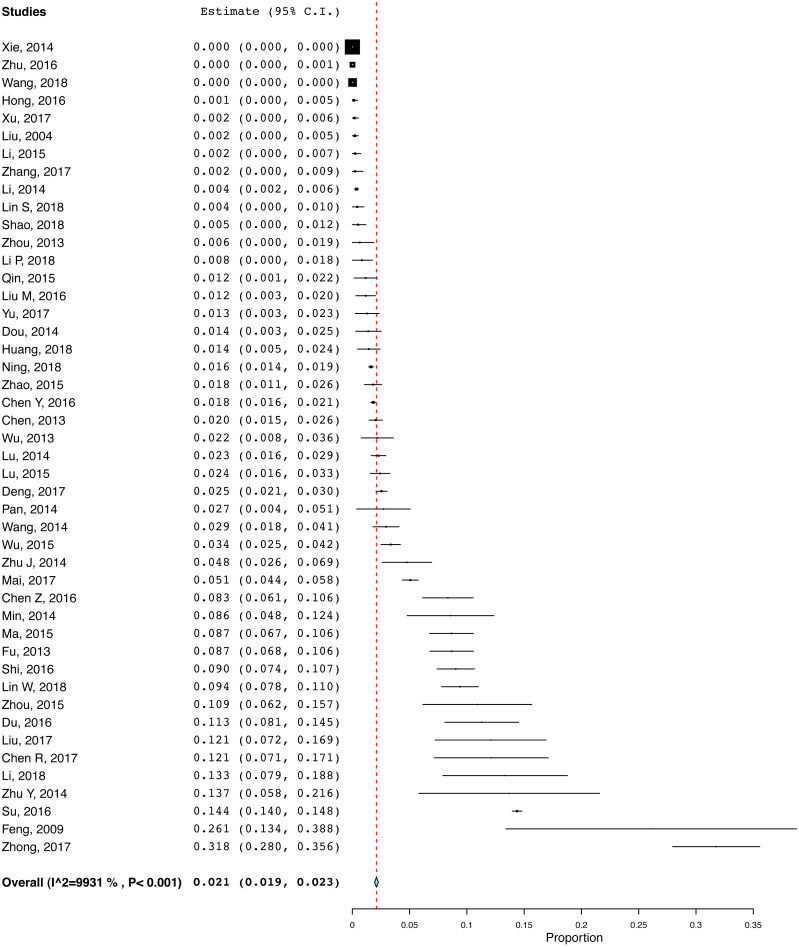
Pooled HIV prevalence of the included studies.

The pooled prevalence of HIV infection in older adults was 2.1% (95% CI [1.9%–2.3%], *I*^2^ = 99.3%, Fig. 2). The results of the subgroup analyses are presented in Table 2. MSM population, hospital population samples, publications after 2014, and studies conducted in the western region were significantly associated with higher HIV infection rate. Meta-regression analyses revealed that higher study quality was significantly associated with higher HIV prevalence (*β* = 0.84, *p* < 0.001, Fig. S1). Meta-regression analysis did not find any significant association between study periods and the prevalence of HIV infection (*β* = −0.03, *p* = 0.76).

### Publication bias

Although funnel plot was visually asymmetrical (Fig. S2), Begg’s test did not find statistically significant publication bias (*p* = 0.21). The Duval and Tweedie trim-and-fill analysis suggests that 8 studies would need to be imputed to achieve an approximate normal error distribution. Including these 8 studies could lead to a lower prevalence of 0.096 (95% CI [0.094–0.099]).

**Table 1:** Characteristics of studies included in this meta-analysis.

	The first author (year)	References	Province	ER	Rural/ Urban	Study period	Study site	Sample size	Mean Age (yrs)	Male (%)	Education (primary school or illiterate %)	Occupation (farmer;%)	Transmission route	Older adults defined age	Study quality
1	Shao, 2018	Shao et al. (2018)	Chongqing	W	Rural	04/2017–05/2017	Community	400	67	100	59%	NR	NR	≥60	5
2	Lin, 2018	Lin, Wu & Zheng (2018)	Hainan	E	Urban	01/2011–12/2016	Hospital	1257	61.5	74.14	NR	52.94	NR	≥50	4
3	Lin, 2018	Lin et al. (2018)	Guangxi	W	Rural	01/2016–08/2016	Community	468	NR	48.5	NR	97.01	NR	≥50	6
4	Li, 2018	Li et al. (2018a)	Qinghai	W	Urban	NR	Community	150	NR	100	NR	NR	NR	≥50	5
5	Huang, 2018	Huang et al. (2018)	Guangxi	W	Rural	08/2016–03/2017	Community	553	NR	49.55	62.39%	97.29	NR	≥50	5
6	Ning, 2018	Ning et al. (2018)	Shanghai	E	Urban	01/2008–12/2014	Community	12910	59.3	100	NR	NR	NR	≥50	5
7	Zhong, 2017	Zhong et al. (2017)	Sichuan	W	Rural	2014–2016	Hospital	576	NR	NR	NR	NR	NR	≥50	5
8	Zhang, 2017	Chen et al. (2017)	Jiangsu	E	Both	10/2015–03/2016	Community	200	68.42	100	73.50%	NR	NR	≥50	5
9	Yu, 2017	Yu (2017)	Guangxi	W	Both	10/2015–01/2016	Community	461	58.82	100	22.30%	10.4	Commercial sex	≥50	6
10	Xu, 2017	Xu & Zhu (2017)	Guangdong	E	Urban	01/2016–04/2016	Community	528	NR	100	NR	NR	NR	≥50	5
11	Maiwulani, 2017	Maiwulani et al. (2017)	Xinjiang	W	Urban	01/2015–12/2015	Hospital	3830	NR	54.31	41.90%	NR	NR	≥50	5
12	Liu, 2017	Liu et al. (2017)	Jiangsu	E	Urban	04/2013–06/2015	Hospital	174	57.15	100	11.50%	NR	NR	≥50	4
13	Deng, 2017	Deng et al. (2017)	Guangxi	W	both	10/2012–12/2012	Community	4048	NR	100	NR	NR	Commercial sex	≥50	5
14	Chen, 2017	Chen et al. (2017)	Guangdong	E	Urban	2010–2016	Hospital	165	NR	NR	NR	NR	NR	≥50	5
15	Zhu, 2016	Zhu et al. (2016)	Jiangsu	E	Rural	NR	Community	2860	71.75	48.39	90.07%	NR	NR	≥65	6
16	Su, 2016	Su et al. (2016)	Yunnan	W	Both	01/2011–12/2013	Hospital	26807	NR	60.3	60.40%	NR	NR	≥50	5
17	Shi, 2016	Shi et al. (2016)	Jiangsu	E	Both	2010–2014	Community	1185	NR	100	52.83%	NR	NR	≥50	5
18	Liu, 2016	Liu et al. (2016)	Hubei	M	both	12/2014–12/2014	Community	601	65.7	100	59.80%	61.50%	NR	≥50	6
19	Hong, 2016	Hong, Zhang & Zhang (2016)	Zhejiang	E	both	03/2014–08/2014	Community	400	64.1	100	73.50%	NR	NR	≥50	4
20	Du, 2016	Du & Meng (2016)	Sichuan	W	both	01/2009–12/2014	Community	372	NR	NR	NR	NR	NR	≥50	4
21	Chen Z, 2016	Chen et al. (2016b)	Chongqing	W	Urban	04/2015–05/2015	Community	599	NR	40.23	29.55%	13.40%	NR	≥60	6
22	Chen Y, 2016	Chen et al. (2016a)	Guangxi	W	both	2010–2015	Community	14105	NR	100	NR	NR	Commercial sex	≥50	4
23	Zhou, 2015	Zhou et al. (2015)	Shanghai	E	Urban	05/2011–04/2013	Community	165	58.7	100	NR	NR	NR	≥50	5
24	Wu, 2015	Wu et al. (2015)	Guangxi	W	both	10/2012–04/2013	Community	1761	64.23	100	74.50%	76.2	Commercial sex	≥50	4
25	Qin, 2015	Qin et al. (2015)	Guangxi	W	Both	2012	Community	430	61.06	100	75.80%	90	Commercial sex	≥50	4
26	Ma, 2015	Ma et al. (2015)	Chongqing	W	Urban	01/2010–12/2014	Hospital	832	60.1	69.11	37.86%	43.51	NR	≥50	4
27	Lu, 2015	Lu et al. (2015)	Guangxi	W	both	10/2012–04/2013	Community	1236	68.42	100	86.92%	88.11	Commercial sex	≥60	4
28	Li, 2015	Li et al. (2015)	Jiangxi	M	NR	01/2013–12/2013	Community	405	NR	65.68	56.30%	NR	NR	≥50	6
29	Zhu Y, 2014	Zhu, Miu & Zhang (2014)	Yunnan	W	NR	01/2013–12/2013	Community	73	NR	NR	NR	NR	NR	≥50	5
30	Zhu J, 2014	Zhu et al. (2014)	Guangxi	W	both	NR	Community	377	61.5	100	73.80%	85.4	Commercial sex	≥50	5
31	Wang, 2014	Wang (2014)	Guangxi	W	both	10/2012–04/2014	Community	848	56.6	100	80.20%	82.7	Commercial sex	≥50	5
32	Min, 2014	Min et al. (2014)	Yunnan	W	both	NR	Community	210	62	100	NR	NR	sex	≥50	4
33	Lu, 2014	Lu et al. (2014)	Guangxi	W	both	10/2012–04/2013	Community	2056	62.28	100	75.73%	78.79	Commercial sex	≥50	4
34	Li, 2014	Li, Wu & Chen (2014)	Zhejiang	E	NR	2006–2012	Community	3860	NR	NR	NR	NR	NR	≥50	5
35	Dou, 2014	Dou (2014)	Anhui	E	Urban	2010–2014	Hospital	427	62.34	100	NR	NR	NR	≥50	5
36	Xie, 2014	Xie et al. (2014)	Zhejiang	E	NR	03/2012–08/2012	Community	215441	63.51	42.26	NR	NR	NR	≥50	6
37	Zhou, 2013	Zhou et al. (2013)	Shanghai	E	Urban	03/2011–09/2011	Hospital	157	60.1	100	15.90%	NR	NR	≥50	5
38	Chen, 2013	Chen et al. (2013)	Guangxi	W	both	04/2012–07/2012	Community	2305	NR	100	NR	NR	Commercial sex	≥50	4
39	Feng, 2009	Feng et al. (2009)	Chongqing	W	NR	07/2006–09/2006, 07/2007–09/2007	Community	46	NR	100	NR	NR	NR	>50	4
40	Liu, 2004	Liu et al. (2004)	Hubei	M	both	01/1999–04/2002	Hospital	902	NR	NR	NR	NR	NR	≥60	5
41	Zhao, 2015	Zhao, Su & Jiang (2015)	Liaoning	M	NR	01/2011–12/2013	Hospital	1217	NR	NR	NR	NR	NR	≥50	4
42	Wu, 2013	Wu et al. (2013)	Guangxi	W	NR	NR	Community	414	NR	100	77.05%	96.14	Commercial sex	≥50	4
43	Li, 2018	Li et al. (2018b)	Sichuan	W	NR	04/2014–12/2015	Community	363	NR	100	NR	NR	Commercial sex	≥50	4
44	Fu, 2013	Fu et al. (2013)	Yunnan	W	NR	01/2008–02/2013	Hospital	842	59.35	64.75	65.82%	74.11	NR	≥50	4
45	Pan, 2014	Pan, Xie & Xu (2014)	Guangdong	E	NR	06/2011–05/2013	Hospital	184	NR	65.21	NR	NR	NR	≥50	4
46	Wang, 2018	Wang et al. (2018a),Wang et al. (2018b)	Henan	M	NR	01/2013–12/2015	Hospital	56199	NR	52.22	NR	NR	NR	≥50	4

**Notes.**

ER Economic Regions E East M Middle W West U Urban R Rural NR Not recorded

## Discussion

This was the first meta-analysis to examine the prevalence of HIV infection in older adults in China. The meta-analysis revealed that the pooled prevalence of HIV infection in older Chinese adults was 2.1%, which was substantially higher than the figure reported in the Chinese general population (0.05%) (National Bureau of Statistics of China, 2018). The high HIV infection rate could be due to several reasons. The life expectancy of HIV-infected adults has been significantly prolonged due to widespread use of HAART (Bhaskaran et al., 2008; Greenbaum et al., 2008; Xing et al., 2014); e.g., the National Free Antiretroviral Treatment Program (NFATP) has covered more than 97% of HIV-infected people in China (Zhang et al., 2009). The high HAART adherence rate in Chinese HIV patients, as confirmed by a recent meta-analysis (Wang et al., 2018b), would be expected to increase life expectancy and many patients are living into their older adulthood. Furthermore, many studies have indicated increasing transmission via commercial sexual activities among older Chinese men after retirement as a major reason for HIV infection (Wang et al., 2014; Zhou et al., 2014). It has been suggested that prevention of HIV transmission among older MSM should be an urgent priority in China’s HIV/AIDS strategy (Ning et al., 2018).

The high proportion of HIV infection in older adults is growing major public health challenge in China. Compared to younger adults, physical and psychiatric comorbidities, such as pneumonia, depression and insomnia, are usually more common in HIV-infected older adults (Ding et al., 2017), which could lead to heavy personal, family and economic burden. Therefore, appropriate allocation of healthcare resources and developing effective preventive strategies for HIV-infected older adults in China should be considered (Xing et al., 2014).

**Table 2:** Subgroup analysis of HIV prevalence in older adults in China.

Subgroup	Number of studies	Sample size	Number of cases	Prevalence (%)	95% CI	*I*^2^ (% with *P*-value)
1. Sexual orientation: MSM population	5	1,720	178	11.8	8.5–15	59.24 (<0.05)
Not specified	41	361,679	5,560	2.0	1.8–2.2	99.4 (<0.001)
2. Sex: Male predominance (≥60%)	32	76,284	5,159	4.1	2.8–5.4	99.2 (<0.001)
No predominance	4	7,711	204	1.7	0.4–3.9	98.6 (<0.001)
3. Study site: Hospital	14	93,569	4,587	7.5	4.5–10.4	99.8 (<0.001)
Community	32	269,830	1,151	1.5	1.3–1.8	97.4 (<0.001)
4. Route of transmission: Commercial sex	12	28,404	610	2.2	1.8–2.6	71.3 (<0.001)
Not specified	33	334,785	5,110	2.0	1.8–2.2	99.5 (<0.001)
5. Education: Primary school and below (≥60%)	13	38,784	4,130	3.4	0.6–6.1	99.7 (<0.001)
Above primary school (≥60%)	8	7,167	205	3.9	2.1–5.8	96.8 (<0.001)
6. Occupation: Farmer predominance (≥60%)	11	9,586	283	2.6	1.7–3.5	90.2 (<0.001)
No predominance	4	3,149	246	6.9	0.2–11.5	97.0 (<0.001)
7. Economic Region: West	26	64,162	5,152	6.0	4.2–7.9	99.3 (<0.001)
Middle	5	59, 324	46	0.5	0.0–1.0	87.1 (<0.001)
East	15	239,913	540	1.0	0.7–1.3	97.5 (<0.001)
8. Area: Rural	5	4, 857	195	4.8	2.6–7.1	98.6 (<0.001)
Urban	12	21,194	732	5.8	4.3–7.3	97.2 (<0.001)
Urban and Rural	18	58,304	4,630	3.8	1.8–5.7	99.5 (<0.001)
9. Defined age for older adults (years): ≥50	41	357,402	5,654	2.4	2.2–2.5	99.4 (<0.001)
≥60	5	5,997	84	1.4	0.6–2.3	95.4 (<0.001)
10. Publication year^a^: In or before 2014	15	22,8142	305	2.1	1.5–2.7	95.4 (<0.001)
After 2014	31	135,257	5,433	3.6	3.1–4.1	99.5 (<0.001)

**Notes.**

a Analyzed using a median splitting method.

Consistent with previous findings on HIV prevalence in China (Zhang et al., 2013), we found in this meta-analysis that MSM was associated with a higher risk of HIV infection; the HIV prevalence in MSM older adults was 11.8%, which was the highest among all subgroups. Compared to community populations, hospital population samples were significantly associated with higher HIV infection rate, which is probably because older adults with HIV infection were more likely to receive HIV testing than those in the community. Compared to middle and eastern economic regions, HIV infection prevalence in older adults was significantly higher in the western region of China, being less developed than other parts of China. Therefore, the lack of access to HIV treatment and prevention measures in the western region could be associated with higher HIV infection rate. Due to degradation of traditional Chinese family structure and lack of family support, retired older men are also more likely to have engaged in commercial sex, particularly in under-developed western regions of China (Huang, Maman & Pan, 2012).

Unexpectedly, commercial sex as the transmission route was not significantly associated with higher HIV infection rate. This appears inconsistent with previous findings that commercial sex is a major route for HIV infection transmission among older Chinese men (Wang et al., 2014; Zhou et al., 2014). Studies published after 2014 were significantly associated with higher HIV infection rate, which we were unable to explain adequately. However, we found that higher study quality was significantly associated with higher HIV prevalence. Due to sigma and discrimination associated with HIV/AIDS, many sufferers, particularly older adults in China usually deny or conceal their diagnosis in order to avoid “loss of face”. High quality studies may identify patients more systematically, and obtain a more accurate and often higher rate HIV infection.

There were several limitations in this meta-analysis. First, similar to other meta-analyses of epidemiological studies (Liu et al., 2016; Long et al., 2014; Wang et al., 2018b; Winsper et al., 2013), there was substantial heterogeneity, although subgroup analyses were performed. The heterogeneity may be associated with different sampling methods, study designs, diagnostic criteria of HIV infection and demographic and clinical characteristics between studies. Second, most studies did not report the transmission route, therefore further sophisticated analyses could not be conducted. Third, due to the cross-sectional design of included studies, the causal relationship between HIV infection and related variables could not be explored.

In conclusion, this meta-analysis showed that the prevalence of HIV infection in older adult population is significantly higher than the general population in China. Attention should be given to this urgent public health issue, and effective HIV/AIDS preventive, screening and treatment measures are warranted in this population.

##  Supplemental Information

10.7717/peerj.9731/supp-1 Supplemental Information 1 Meta-regression for study quality and HIV prevalence

10.7717/peerj.9731/supp-2 Supplemental Information 2 Funnel plot of standard error by logit event rate

10.7717/peerj.9731/supp-3 Supplemental Information 3 Quality assessment for included studies

10.7717/peerj.9731/supp-4 Supplemental Information 4 Rationale and contribution

10.7717/peerj.9731/supp-5 Supplemental Information 5 PRISMA checklist

## Additional Information and Declarations

### Data Availability

The following information was supplied regarding data availability: The collated data from this systematic review are available in the Tables.
