# Supplementary material for: Older adults at high risk of HIV infection in China: a systematic review and meta-analysis of observational studies
Source: PeerJ. 2020 Oct 21;8:e9731. doi: 10.7717/peerj.9731 (PMC7585370; doi:10.7717/peerj.9731)
Supplement: Supplemental Information 1 [file peerj-08-9731-s001.docx]

Supplementary 1. Meta- regression for study quality and HIV prevalence.
