# Supplementary material for: Older adults at high risk of HIV infection in China: a systematic review and meta-analysis of observational studies
Source: PeerJ. 2020 Oct 21;8:e9731. doi: 10.7717/peerj.9731 (PMC7585370; doi:10.7717/peerj.9731)
Supplement: Supplemental Information 3 [file peerj-08-9731-s003.docx]

Supplementary 3. Quality assessment

|  |  | Is the target population clearly defined | Was either of the following ascertainment methods used ?(1) probability sampling, or (2) entire population surveyed | Is the response rate >70% | Are non-responders clearly described? | Is the sample representative of the target population? | Were data collection methods standardized? | Were validated criteria used to assess for the presence/absence of disease? | Are the estimates of prevalence given with confidence intervals and in detail by subgroup (if applicable)? | **total** |
| --- | --- | --- | --- | --- | --- | --- | --- | --- | --- | --- |
| 1 | Shao, 2018 | 1 | 1 | 0 | 0 | 1 | 1 | 1 | 0 | 5 |
| 2 | Lin W, 2018 | 1 | 0 | 0 | 0 | 1 | 1 | 1 | 0 | 4 |
| 3 | Lin S, 2018 | 1 | 1 | 1 | 0 | 1 | 1 | 1 | 0 | 6 |
| 4 | Li, 2018 | 1 | 1 | 0 | 0 | 1 | 1 | 1 | 0 | 5 |
| 5 | Huang, 2018 | 1 | 0 | 1 | 0 | 1 | 1 | 1 | 0 | 5 |
| 6 | Ning, 2018 | 1 | 0 | 0 | 0 | 1 | 1 | 1 | 1 | 5 |
| 7 | Zhong, 2017 | 1 | 1 | 0 | 0 | 1 | 1 | 1 | 0 | 5 |
| 8 | Zhang, 2017 | 1 | 1 | 0 | 0 | 1 | 1 | 1 | 0 | 5 |
| 9 | Yu, 2017 | 1 | 1 | 1 | 0 | 1 | 1 | 1 | 0 | 6 |
| 10 | Xu, 2017 | 1 | 0 | 1 | 0 | 1 | 1 | 1 | 0 | 5 |
| 11 | Mai, 2017 | 1 | 1 | 0 | 0 | 1 | 1 | 1 | 0 | 5 |
| 12 | Liu, 2017 | 1 | 0 | 0 | 0 | 1 | 1 | 1 | 0 | 4 |
| 13 | Deng, 2017 | 1 | 1 | 0 | 0 | 1 | 1 | 1 | 0 | 5 |
| 14 | Chen R, 2017 | 1 | 1 | 0 | 0 | 1 | 1 | 1 | 0 | 5 |
| 15 | Zhu, 2016 | 1 | 1 | 1 | 0 | 1 | 1 | 1 | 0 | 6 |
| 16 | Su, 2016 | 1 | 1 | 0 | 0 | 1 | 1 | 1 | 0 | 5 |
| 17 | Shi, 2016 | 1 | 1 | 0 | 0 | 1 | 1 | 1 | 0 | 5 |
| 18 | Liu M, 2016 | 1 | 1 | 1 | 0 | 1 | 1 | 1 | 0 | 6 |
| 19 | Hong, 2016 | 1 | 0 | 0 | 0 | 1 | 1 | 1 | 0 | 4 |
| 20 | Du, 2016 | 1 | 0 | 0 | 0 | 1 | 1 | 1 | 0 | 4 |
| 21 | Chen Z, 2016 | 1 | 1 | 1 | 0 | 1 | 1 | 1 | 0 | 6 |
| 22 | Chen Y, 2016 | 1 | 0 | 0 | 0 | 1 | 1 | 1 | 0 | 4 |
| 23 | Zhou, 2015 | 1 | 1 | 0 | 0 | 1 | 1 | 1 | 0 | 5 |
| 24 | Wu, 2015 | 1 | 0 | 0 | 0 | 1 | 1 | 1 | 0 | 4 |
| 25 | Qin, 2015 | 1 | 0 | 0 | 0 | 1 | 1 | 1 | 0 | 4 |
| 26 | Ma, 2015 | 1 | 0 | 0 | 0 | 1 | 1 | 1 | 0 | 4 |
| 27 | Lu, 2015 | 1 | 0 | 0 | 0 | 1 | 1 | 1 | 0 | 4 |
| 28 | Li, 2015 | 1 | 1 | 1 | 0 | 1 | 1 | 1 | 0 | 6 |
| 29 | Zhu Y, 2014 | 1 | 1 | 0 | 0 | 1 | 1 | 1 | 0 | 5 |
| 30 | Zhu J, 2014 | 1 | 1 | 0 | 0 | 1 | 1 | 1 | 0 | 5 |
| 31 | Wang, 2014 | 1 | 0 | 1 | 0 | 1 | 1 | 1 | 0 | 5 |
| 32 | Min, 2014 | 1 | 0 | 0 | 0 | 1 | 1 | 1 | 0 | 4 |
| 33 | Lu, 2014 | 1 | 0 | 0 | 0 | 1 | 1 | 1 | 0 | 4 |
| 34 | Li, 2014 | 1 | 1 | 0 | 0 | 1 | 1 | 1 | 0 | 5 |
| 35 | Dou, 2014 | 1 | 1 | 0 | 0 | 1 | 1 | 1 | 0 | 5 |
| 36 | Xie, 2014 | 1 | 1 | 1 | 0 | 1 | 1 | 1 | 0 | 6 |
| 37 | Zhou, 2013 | 1 | 1 | 0 | 0 | 1 | 1 | 1 | 0 | 5 |
| 38 | Chen, 2013 | 1 | 0 | 0 | 0 | 1 | 1 | 1 | 0 | 4 |
| 39 | Feng, 2009 | 1 | 0 | 0 | 0 | 1 | 1 | 1 | 0 | 4 |
| 40 | Liu, 2004 | 1 | 1 | 0 | 0 | 1 | 1 | 1 | 0 | 5 |
| 41 | Zhao, 2015 | 1 | 0 | 0 | 0 | 1 | 1 | 1 | 0 | 4 |
| 42 | Wu, 2013 | 1 | 0 | 0 | 0 | 1 | 1 | 1 | 0 | 4 |
| 43 | Li P, 2018 | 1 | 0 | 0 | 0 | 1 | 1 | 1 | 0 | 4 |
| 44 | Fu, 2013 | 1 | 0 | 0 | 0 | 1 | 1 | 1 | 0 | 4 |
| 45 | Pan, 2014 | 1 | 0 | 0 | 0 | 1 | 1 | 1 | 0 | 4 |
| 46 | Wang, 2018 | 1 | 0 | 0 | 0 | 1 | 1 | 1 | 0 | 4 |
