# Supplementary material for: Older adults at high risk of HIV infection in China: a systematic review and meta-analysis of observational studies
Source: PeerJ. 2020 Oct 21;8:e9731. doi: 10.7717/peerj.9731 (PMC7585370; doi:10.7717/peerj.9731)
Supplement: Supplemental Information 4 [file peerj-08-9731-s004.docx]

Rationale:

In China, the incidence of HIV was 4.1 per 100,000 individuals in 2018, which translates to approximately 56,993 newly infected persons (National Bureau of Statistics of China 2018). Those who are at high risk of HIV infection include men who have sex with men (MSM), and there has been an increasing prevalence in older adults recently. In order to develop appropriate strategies for HIV prevention and control, it is important to accurately determine the prevalence of HIV infection. In order to develop appropriate strategies for HIV prevention and control, it is important to accurately determine the prevalence of HIV infection.

Contribution:

This was the first meta-analysis to examine the prevalence of HIV infection in older adults in China. The meta-analysis revealed that the pooled prevalence of HIV infection in older Chinese adults was 3.3%, which was 66 times higher than the figure reported in the Chinese general population (0.05%).
