# Supplementary material for: Older adults at high risk of HIV infection in China: a systematic review and meta-analysis of observational studies
Source: PeerJ. 2020 Oct 21;8:e9731. doi: 10.7717/peerj.9731 (PMC7585370; doi:10.7717/peerj.9731)
Supplement: Supplemental Information 5 [file peerj-08-9731-s005.docx]

| **Section/topic** | **#** | **Checklist item** | **Reported on page #** |
| --- | --- | --- | --- |
| **TITLE** | | | Page 1 |
| Title | 1 | Identify the report as a systematic review, meta-analysis, or both. | Page 1 “Older adults at high risk of HIV infection in China: a systematic review and meta-analysis of observational studies  “ |
| **ABSTRACT** | | | Page 3 |
| Structured summary | 2 | Provide a structured summary including, as applicable: background; objectives; data sources; study eligibility criteria, participants, and interventions; study appraisal and synthesis methods; results; limitations; conclusions and implications of key findings; systematic review registration number. | Page 3 “The prevalence of human immunodeficiency virus (HIV) infection in older adults in China is increasing, but the findings across prevalence studies have been mixed. This is the first meta-analysis of the prevalence of HIV infection and its moderating factors in older adults in China. Two investigators systematically and independently searched both international (PubMed, PsycINFO, Web of Sciences, and EMBASE) and Chinese (WanFang, CNKI, and CQVIP) databases. HIV infection rates in older adults were analyzed using a random-effects model. Altogether 46 studies were included in the analysis, with 363,399 subjects (including 5,738 with HIV infection). The pooled prevalence of HIV infection in older adults was 2.1% (95%CI: 1.9%-2.3%, I^2^ = 99.3%). Subgroup analyses revealed that men who have sex with men (MSM), hospital population samples, publications after 2014, and studies conducted in the western region were significantly associated with higher rate of HIV infection. This meta-analysis found that the HIV infection prevalence in older adults is significantly higher than the general population in China. Attention should be given to this urgent public health issue, and effective HIV/AIDS preventive, screening and treatment measures are warranted in this population.”  **PROSPERO:** CRD42019124286 |
| **INTRODUCTION** | | |  |
| Rationale | 3 | Describe the rationale for the review in the context of what is already known. | Page 4 “Epidemiological studies in China has found an increasing trend in number and proportion of HIV infection among older adults” |
| Objectives | 4 | Provide an explicit statement of questions being addressed with reference to participants, interventions, comparisons, outcomes, and study design (PICOS). | Page 6 “ The inclusion criteria were studies that: (1) reported prevalence of HIV infection in older adults or provided information to calculate the prevalence. The diagnosis of HIV infection was based on study-defined criteria; (2) were cross-sectional or cohort studies (only baseline data were included) with meta-analyzable data; (3) were conducted in older adults (i.e., aged 50 years and older) in China. Case series, reviews, and meta-analyses were excluded.“ |
| **METHODS** | | |  |
| Protocol and registration | 5 | Indicate if a review protocol exists, if and where it can be accessed (e.g., Web address), and, if available, provide registration information including registration number. | Page 3 “This meta-analysis was performed according to the Preferred Reporting Items for Systematic Review and Meta-Analyses (PRISMA) (Moher et al. 2009)**.** Both International (PubMed, PsycINFO, Web of Science, and EMBASE) and Chinese (WanFang, CNKI and CQVIP) databases were systematically and independently searched by two investigators (Yuan Yang and Chang Chen) from their inception date up to May 1, 2020. The following search terms were used: (“HIV” OR “AIDS” OR “human immunodeficiency virus” OR “acquired immune deficiency syndrome”) AND (“epidemiology” OR “prevalence” OR “rate” OR “proportion”) AND (“old” OR “older” OR “elderly” OR “aged” OR “aging”) AND (“Chinese” OR “China”).” |
| Eligibility criteria | 6 | Specify study characteristics (e.g., PICOS, length of follow-up) and report characteristics (e.g., years considered, language, publication status) used as criteria for eligibility, giving rationale. | Page 7  “(1) reported prevalence of HIV infection in older adults or provided information to calculate the prevalence. The diagnosis of HIV infection was based on study-defined criteria; (2) were cross-sectional or cohort studies (only baseline data were included) with meta-analyzable data; (3) were conducted in older adults (i.e., aged 50 years and older) in China. Case series, reviews, and meta-analyses were excluded.” |
| Information sources | 7 | Describe all information sources (e.g., databases with dates of coverage, contact with study authors to identify additional studies) in the search and date last searched. | Page 6  “Both International (PubMed, PsycINFO, Web of Science, and EMBASE) and Chinese (WanFang, CNKI and CQVIP) databases were systematically and independently searched by two investigators (Yuan Yang and Chang Chen) from their inception date up to May 1, 2020.” |
| Search | 8 | Present full electronic search strategy for at least one database, including any limits used, such that it could be repeated. | Page 6  “Both International (PubMed, PsycINFO, Web of Science, and EMBASE) and Chinese (WanFang, CNKI and CQVIP) databases were systematically and independently searched by two investigators (Yuan Yang and Chang Chen) from their inception date up to May 1, 2020.” |
| Study selection | 9 | State the process for selecting studies (i.e., screening, eligibility, included in systematic review, and, if applicable, included in the meta-analysis). | Page 6  “two investigators (Yuan Yang and Chang Chen) from their inception date up to May 1, 2020. Reference lists of eligible studies and relevant review articles were also hand-searched.  ” |
| Data collection process | 10 | Describe method of data extraction from reports (e.g., piloted forms, independently, in duplicate) and any processes for obtaining and confirming data from investigators. | Page 7  “Systematic literature search and data extraction were independently conducted by two investigators. The titles and abstracts of potential publications were screened separately by two reviewers (Yuan Yang and Chang Chen) before the full texts were read for eligibility. Any inconsistencies in the process were discussed and resolved by a third reviewer (Yuan-Yuan Wang). The following information was extracted: year of publication, survey period, study site, sampling method, sample size and response rate, mean age, sex, education, occupation, province, rural or urban area, definition of older adults (e.g., above 50 years), and transmission route (e.g., commercial sex). “ |
| Data items | 11 | List and define all variables for which data were sought (e.g., PICOS, funding sources) and any assumptions and simplifications made. | Page 7  The following information was extracted: year of publication, survey period, study site, sampling method, sample size and response rate, mean age, sex, education, occupation, province, rural or urban area, definition of older adults (e.g., above 50 years), and transmission route (e.g., commercial sex). |
| Risk of bias in individual studies | 12 | Describe methods used for assessing risk of bias of individual studies (including specification of whether this was done at the study or outcome level), and how this information is to be used in any data synthesis. | Page 7-8  Two investigators independently evaluated the methodological assessment, using the critical appraisal for epidemiological studies (Loney et al. 1998) that contains 8 items covering three aspects: sampling, measurement and analysis. The total score of this instrument is 8; the total score of 7-8 was considered as ‘high quality’, 4-6 as ‘moderate quality’, and 0-3 as ‘low quality’ (Loney et al. 1998). Any inconsistencies were resolved by a discussion with a third investigator. |
| Summary measures | 13 | State the principal summary measures (e.g., risk ratio, difference in means). | Page 7-8  Due to different sampling methods, study designs and demographic and clinical characteristics between studies, the random-effects model was used (DerSimonian & Laird 1986). The heterogeneity of outcomes were assessed using I^2^, with I^2^ > 50% as significant heterogeneity (Higgins et al. 2003). Following the recommendation of the Cochrane handbook (Higgins et al. 2019) and other studies (Li et al. 2020; Xu et al. 2020; Yang et al. 2020), publication bias was assessed using the funnel plots and Begg ’s test and Tweedie’s trim-and-fill analysis. All data analyses were 2 tailed, and the significant level was set at 0.05.  Subgroup and meta-regression analyses were performed to examine the moderating factors of HIV infection prevalence. |
| Synthesis of results | 14 | Describe the methods of handling data and combining results of studies, if done, including measures of consistency (e.g., I^2^) for each meta-analysis. | Page 7-8  The heterogeneity of outcomes were assessed using I^2^, with I^2^ > 50% as significant heterogeneity (Higgins et al. 2003). Following the recommendation of the Cochrane handbook (Higgins et al. 2019) and other studies (Li et al. 2020; Xu et al. 2020; Yang et al. 2020), publication bias was assessed using the funnel plots and Begg ’s test and Tweedie’s trim-and-fill analysis. |

Page 1 of 2

| **Section/topic** | **#** | **Checklist item** | **Reported on page #** |
| --- | --- | --- | --- |
| Risk of bias across studies | 15 | Specify any assessment of risk of bias that may affect the cumulative evidence (e.g., publication bias, selective reporting within studies). | Page 7-8  Although funnel plot was visually asymmetrical (Supplemental Figure 2), Begg’s test did not find statistically significant publication bias (p = 0.21). The Duval and Tweedie trim-and-fill analysis suggests that 8 studies would need to be imputed to achieve an approximate normal error distribution. Including these 8 studies could lead to a lower prevalence of 0.096 (95%CI: 0.094 to 0.099). |
| Additional analyses | 16 | Describe methods of additional analyses (e.g., sensitivity or subgroup analyses, meta-regression), if done, indicating which were pre-specified. | Page 8-9  Meta-regression analyses revealed that higher study quality was significantly associated with higher HIV prevalence (β = 0.84, p< 0.001, Supplemental Figure 1). Meta-regression analysis did not find any significant association between study periods and the prevalence of HIV infection (β = -0.03, p = 0.76). |
| **RESULTS** | | |  |
| Study selection | 17 | Give numbers of studies screened, assessed for eligibility, and included in the review, with reasons for exclusions at each stage, ideally with a flow diagram. | Page 9  See Figure 1 |
| Study characteristics | 18 | For each study, present characteristics for which data were extracted (e.g., study size, PICOS, follow-up period) and provide the citations. | Table 1, page 10 |
| Risk of bias within studies | 19 | Present data on risk of bias of each study and, if available, any outcome level assessment (see item 12). | Page 10-11  See Supplementary figure 2 |
| Results of individual studies | 20 | For all outcomes considered (benefits or harms), present, for each study: (a) simple summary data for each intervention group (b) effect estimates and confidence intervals, ideally with a forest plot. | Page 10-11  See Table 2 |
| Synthesis of results | 21 | Present results of each meta-analysis done, including confidence intervals and measures of consistency. | See Figure 1 |
| Risk of bias across studies | 22 | Present results of any assessment of risk of bias across studies (see Item 15). | Supplementary table 3 |
| Additional analysis | 23 | Give results of additional analyses, if done (e.g., sensitivity or subgroup analyses, meta-regression [see Item 16]). | Page 9-10  The pooled prevalence of HIV infection in older adults was 2.1% (95%CI: 1.9%- 2.3%, I^2^ = 99.3%, Figure 2). The results of the subgroup analyses are presented in Table 2. MSM population, hospital population samples, non-specified transmission route, publications after 2014, and studies conducted in the western region were significantly associated with higher HIV infection rate. Meta-regression analyses revealed that higher study quality was significantly associated with higher HIV prevalence (β = 0.84, p< 0.001, Supplemental Figure 1). |
| **DISCUSSION** | | |  |
| Summary of evidence | 24 | Summarize the main findings including the strength of evidence for each main outcome; consider their relevance to key groups (e.g., healthcare providers, users, and policy makers). | Page 11-12  This was the first meta-analysis to examine the prevalence of HIV infection in older adults in China. The meta-analysis revealed that the pooled prevalence of HIV infection in older Chinese adults was 2.1%, which was substantially higher than the figure reported in the Chinese general population (0.05%) (National Bureau of Statistics of China 2018). |
| Limitations | 25 | Discuss limitations at study and outcome level (e.g., risk of bias), and at review-level (e.g., incomplete retrieval of identified research, reporting bias). | Page 13  There were several limitations in this meta-analysis. First, similar to other meta-analysis of epidemiological studies (Liu et al. 2016; Long et al. 2014; Wang et al. 2018b; Winsper et al. 2013), there is substantial heterogeneity, although subgroup analyses were performed. The heterogeneity may be associated with different sampling methods, study designs, diagnostic criteria of HIV infection and demographic and clinical characteristics between studies. Second, most studies did not report the transmission route, therefore further sophisticated analyses could not be conducted. Third, due to the cross-sectional design of included studies, the causal relationship between HIV infection and related variables could not be explored. |
| Conclusions | 26 | Provide a general interpretation of the results in the context of other evidence, and implications for future research. | Page 13  the meta-analysis showed that the prevalence of HIV infection in older adult population is significantly higher than the general population in China. Attention should be given to this urgent public health issue, and effective HIV/AIDS preventive, screening and treatment measures are warranted in this population. |
| **FUNDING** | | |  |
| Funding | 27 | Describe sources of funding for the systematic review and other support (e.g., supply of data); role of funders for the systematic review. | Page 2  The study was supported by the University of Macau (MYRG2015-00230-FHS; MYRG2016-00005-FHS), the National Key Research & Development Program of China (No. 2016YFC1307200), the Beijing Municipal Administration of Hospitals Clinical Medicine Development of Special Funding Support (No.ZYLX201607) and the Beijing Municipal Administration of Hospitals’ Ascent Plan (No. DFL20151801). |

*From:*  Moher D, Liberati A, Tetzlaff J, Altman DG, The PRISMA Group (2009). Preferred Reporting Items for Systematic Reviews and Meta-Analyses: The PRISMA Statement. PLoS Med 6(7): e1000097. doi:10.1371/journal.pmed1000097

For more information, visit: **www.prisma-statement.org**.

Page 2 of 2
